# Understanding Consumers’ Purchase Intention Towards Meat Produced Without Preventive Antibiotic Use

**DOI:** 10.3390/foods13233779

**Published:** 2024-11-25

**Authors:** Yingnan Zhou, Airong Zhang, Rieks Dekker van Klinken, Junxiu Wang

**Affiliations:** 1 School of Mental Health, Wenzhou Medical University, Wenzhou 325035, China; lesliezhou56@outlook.com; 2 Health and Biosecurity, Commonwealth Scientific and Industrial Research Organisation (CSIRO), Brisbane, QLD 4102, Australia; airong.zhang@csiro.au (A.Z.); rieks.vanklinken@csiro.au (R.D.v.K.); 3 The Affiliated Kangning Hospital of Wenzhou Medical University, Wenzhou 325007, China

**Keywords:** consumer, purchase intention, preventive antibiotic use, food animals

## Abstract

Antibiotics are widely used to prevent healthy animals from getting diseases in livestock industries. Such practice has greatly contributed to the increase in antibiotic-resistant pathogens in animals and in the environment, which poses severe health threats to humans. This study aims to investigate consumers’ purchase intention towards meat produced without preventive antibiotic use, and to identify key factors influencing this purchase intention. An online survey of 1123 participants was conducted in China. Descriptive statistical analysis, correlation, and regression analysis were conducted. The results suggested that consumers had a negative attitude towards preventive antibiotic use in food animals and a strong purchase intention towards meat produced without it. The key drivers of this purchase intention included health consciousness, trust in responsible antibiotic use in farming, objective knowledge about antibiotics used in food animals, subjective knowledge about preventive antibiotic use, concerns over antibiotic residues, and attitude towards preventive antibiotic use. These findings can provide deep insights for policymakers and livestock industries who seek to promote responsible antibiotic use and develop effective communication strategies with consumers.

## 1. Introduction

Antibiotics are among the most important medicines for treating bacterial infections in both humans and food animals [1,2]. However, due to overuse and misuse of antibiotics in various sectors (i.e., humans, agriculture, and aquaculture), more and more bacteria have become resistant to existing antibiotics [3,4]. As a result, many common infectious diseases have become much harder, if not impossible, to treat [5].

Globally, the consumption of antibiotics in animals is about three times more than in humans [6]. Most antibiotic classes considered medically important to human health are widely used in food animals. For example, over 70% of the antibiotics which are clinically important to humans are utilized in livestock in the US, and more than 50% in most countries globally [4]. The main purposes of antibiotic use in food animals are threefold: to treat animals with infectious diseases, to stimulate animal growth, and to prevent animals from becoming sick [2,4,7,8]. Using antibiotics to treat clinically diagnosed bacterial infections is important for maintaining animal health and food safety. However, a large proportion of antibiotics are administered to healthy animals for growth promotion or disease prevention rather than for treating diseases, which represents overuse and misuse [9,10,11]. Antibiotic growth promotion refers to administering low doses of antibiotics to stimulate animal growth or to increase feed efficiency [12]. In recent years, it has been restricted by various countries (e.g., Australia, the EU, Canada, the US, and China) [13,14,15,16]. Using antibiotics to prevent diseases includes metaphylaxis and prophylaxis [12]. Metaphylaxis refers to administering antibiotics to a group of animals after a clinical disease has been diagnosed in part of the group [17], whereas prophylaxis refers to administering antibiotics to healthy animals to prevent diseases before clinical signs appear [17]. The EU has issued new regulations on veterinary medicines to restrict the use of antibiotics for metaphylaxis and prophylaxis. Metaphylaxis is now limited to cases where there is a high risk of infection spreading within a group of animals and no appropriate alternatives are available, while prophylaxis has been banned, with exceptions allowing administration to individual animals in specific cases [17]. The World Health Organization (WHO) has also issued guidelines on the use of antibiotics in food animals, where it was strongly recommended to completely restrict using medically important antibiotics to prevent diseases without clinical diagnosis [18]. Recently, an Australian super fund and Texan nuns lobbied McDonald’s to put restrictions on the use of antibiotics for disease prevention in its supply chain [19]. In addition to these regulatory changes and activism, there is also growing scientific research investigating consumers’ perspectives on this practice [20,21,22]. Hence, this study focuses on the use of antibiotics for prophylaxis. Here onwards, the term preventive antibiotic use refers to prophylaxis.

Preventive antibiotic use is a common farming practice in various countries (e.g., Brazil, Cambodia, Thailand, Vietnam, and China) [23,24,25,26,27,28]. Farmers in these countries mostly use antibiotics for disease prevention rather than treatment [23,28], which is considered a cheap solution to reduce animal mortality [25]. Farmers alternately utilize different antibiotics in a cyclical pattern [23,27,28]. For example, in Thailand, five cycles of medication were given to chickens raised for 41 days from day 1 to day 31, with 4 days of continuous use followed by 2–4 days of no use [27]. Consequently, an individual animal could be subjected to over five different classes of antibiotics throughout its entire life [23,27]. Such preventive use has significantly contributed to the increase in antibiotic-resistant pathogens in animals and in the environment [29,30,31,32]. These antibiotic-resistant pathogens can transmit to humans through various sources (i.e., via contacting animals, food chain, and exposure to resistant pathogens in the environment), thus imposing significant health threats to humans [33,34]. The occurrence and mortality of foodborne diseases is increasing due to the increasing prevalence of antibiotic-resistant foodborne pathogens such as E. coli, Staphylococcus aureus, Listeria monocytogenes, Salmonella, Campylobacter, and Enterococci [35,36,37]. It has been estimated that antibiotic-resistant pathogens directly caused 1.27 million deaths and contributed to 4.95 million deaths in 2019, with antibiotic-resistant E. coli and Staphylococcus aureus infections being the two leading causes [38]. Moreover, the routine use of antibiotics to prevent diseases in food animals can also lead to the presence of antibiotic residues in food, which may cause direct toxic effects in sensitive populations after being consumed [1,2,9].

The overuse and misuse of antibiotics in food animals have drawn consumers’ concerns in various countries [39,40,41]. These increasing concerns have led to the emergence and marketing of animal-derived food produced without any use of antibiotics (e.g., “antibiotic-free” or “raised without antibiotics” products) [42,43,44,45,46]. This trend is problematic, as excluding antibiotics from infection treatments not only endangers animal health but also hinders food safety [47,48]. Therefore, it is important to understand consumers’ perspectives on preventive antibiotic use rather than a complete ban on all usage. So far, limited research has been conducted to investigate consumers’ perspectives on the preventive use, and the findings are fragmented and sometimes contradictory. For instance, studies conducted in Europe and in the US revealed that consumers could accept the use of antibiotics to treat diseases but not to prevent diseases [20,21], while another US study reported that only a minority of participants considered preventive use concerning and unacceptable [22]. Moreover, consumers’ purchase intention towards meat produced without preventive antibiotic use and the underlying factors is poorly understood.

China is one of the largest producers and consumers of antibiotics, where a substantial amount of antibiotics are used in both humans and food animals [49]. It was estimated that a total of 162,000 tons of antibiotics were consumed in China in 2013, with 52% being used in animals [50]. To reduce overuse and misuse of antibiotics in food animals, China has banned antibiotic growth promoters since 2020 [15]. Preventive antibiotic use, however, has not been restricted by legislation in China. Further, Chinese consumers’ perspectives on this practice is less understood. Therefore, this study aims to explore Chinese consumers’ purchase intention towards meat produced without preventive antibiotic use and its drivers.

### 1.1. Literature Review and Hypothesis Develpment

The Theory of Planned Behavior (TPB) has been widely applied to understand consumers’ purchase behaviors [51,52,53,54,55,56,57,58,59]. This model posits that people’s intention to perform a behavior is determined by three key factors: attitude towards the behavior, the perceived social norm regarding whether to engage in the behavior, and perceived behavioral control over performing the behavior [60]. In the context of consumers’ food purchase intentions, a growing body of research has extended this framework by incorporating other variables, including health consciousness, trust, knowledge, and risk perception, leading to the establishment of various extended TPB models, e.g., [53,54,55,56,57,61,62,63,64,65,66]. Informed by this research, we propose that attitude, health consciousness, trust in responsible antibiotic use in farming, knowledge, and risk perception are the potential drivers of consumers’ purchase intention towards meat produced without preventive antibiotic use. The perceived social norm and perceived behavioral control are not included in this study due to the hypothetical nature of the food product being investigated—meat produced without preventive antibiotic use. Since this product does not yet exist in the market, consumers lack real-life exposure or experience with it. As a result, there are no established social norms or tangible behavioral control factors influencing their purchasing decisions.

To inform hypothesis development, we explored the broader literature on consumers’ food purchase decisions that may be applicable to meat produced without preventive antibiotic use. We particularly focused on the literature regarding antibiotic-use-labeled food products (e.g., antibiotic-free) and organic food. Organic food was chosen due to its strict restriction of antibiotic use in production [67,68]. Hence, findings from these lines of research may help identify the key predictors for purchasing meat produced without preventive antibiotic use.

#### 1.1.1. Health Consciousness

Health consciousness has been integrated into the TPB as a key driver of consumers’ food purchase intention [64,65]. Research show that consumers with higher health consciousness are more likely to engage in behaviors which will help maintain or improve their health [69,70]. Health consciousness has been found to be a strong predictor of consumers’ purchase intentions towards various food products, such as organic food [70,71,72], vitamins and nutritional supplements [73], and selenium-rich vegetables [69]. This is because consumers believe that these products are healthier or will deliver health benefits to them [70,74,75]. For instance, an important reason for consumers to purchase organic food is to avoid consuming harmful chemicals (e.g., pesticides, hormones, antibiotics, and food additives) [74,75,76]. Accordingly, it can be inferred that consumers with higher health consciousness are more concerned about the health consequences of preventive antibiotic use in food animals, and thus have higher purchase intention towards meat produced without it. Therefore, Hypothesis 1 is as follows:

**H1:**  *Health consciousness will positively affect purchase intention towards meat produced without preventive antibiotic use*.

#### 1.1.2. Trust in Responsible Antibiotic Use in Farming

Trust is another key variable identified as an underlying factor in understanding consumers’ food purchase decisions by research applying extended TPB models [53,54,66]. For instance, Giampietri et al. found that trust and attitude were both significant predictors of intention to purchase food from short food supply chains [54]. A substantial body of research has shown that consumers’ intention to purchase credence goods, such as organic food, is negatively impacted by their mistrust. This mistrust arises from concerns about the quality and authenticity of these products, the production systems used, and the credibility of the certification and labeling associated with them [66,75,77,78,79]. Conversely, a higher level of trust in these aspects can reduce consumers’ sense of uncertainty and risk perception, as well as increase consumers’ perceived benefits of buying the products, thereby facilitating purchase intention [79,80]. Therefore, we propose that consumers’ trust in responsible antibiotic use in farming plays an important role in affecting purchase intention. When consumers believe that antibiotics are administered to food animals responsibly and in full compliance with regulations, they will have higher trust in the production system and the quality of meat produced without preventive antibiotic use. Thus, Hypothesis 2 is as follows:

**H2:**  *Trust in responsible antibiotic use in farming will positively affect purchase intention towards meat produced without preventive antibiotic use*.

#### 1.1.3. Knowledge

Several studies have integrated knowledge into the TPB to draw a deep understanding of consumers’ food choices [56,57,66]. For example, Mohd Suki and Abang Salleh found that knowledge about the Halal principle positively affected Muslim consumers’ intention to purchase from Halal food retail stores [56].

Two types of knowledge are commonly used in consumer research: objective knowledge (i.e., how much an individual actually knows) and subjective knowledge (i.e., how much an individual thinks they know) [22,81,82]. These two types of knowledge are distinct measures of knowledge and are only weakly to moderately correlated [81,83,84]. Both objective and subjective knowledge are found to positively affect purchase decisions regarding a wide range of products, including organic food [77], environmentally sustainable food [85], imported functional food [86], green products [87], and insect food [88]. With regard to antibiotic use in food animals and antibiotic resistance, Schell et al. found that consumers who self-reported being aware of antibiotic resistance expressed a higher intention to buy milk labeled with responsible antibiotic use [89]. Moreover, Meerza et al. found that consumers, who had a higher level of objective knowledge about antibiotic use in food animals and antibiotic resistance, were more likely to accept the use of antibiotics for disease treatment and control, but less likely to accept their use for disease prevention [22]. These findings suggest that both objective and subjective knowledge can affect consumers’ attitude and purchase intention towards antibiotic use in food animals. Therefore, Hypotheses 3 and 4 are as follows:

**H3:**  *Objective knowledge about antibiotics used in food animals will positively affect purchase intention towards meat produced without preventive antibiotic use*.

**H4:**  *Subjective knowledge about preventive antibiotic use in food animals will positively affect purchase intention towards meat produced without preventive antibiotic use*.

#### 1.1.4. Concerns over Antibiotic Residues and Resistant Bacteria

Previous research has included risk perception in the TPB to examine consumers’ purchase intentions [61,62,63]. This factor is particularly relevant to this study, as the overuse and misuse of antibiotics in food animals pose serious health risks to humans. On one hand, they lead to the presence of antibiotic residues in food. On the other hand, they significantly increase the risk of transmission of antibiotic-resistant bacteria from food animals to humans [1,2]. Consumers are aware of these health risks and are concerned about them [39,40,41]. These concerns have been found to be significant drivers of purchase decisions for food produced with reduced antibiotic use or antibiotic-free food [40,42,61,90]. For instance, by integrating risk perceptions into the TPB, Bradford et al. found that concerns over antibiotic use in food animals and antibiotic resistance significantly influenced purchase intention towards pork with traceable information on antibiotic use [61]. Denver et al. suggested that consumers were willing to pay more for meat with reduced antibiotic use due to a belief that there were less antibiotic residues and resistant bacteria in this meat [40]. Consumers who received information on the health risks of antibiotic residues and resistant bacteria had a higher intention to buy meat produced without preventive antibiotic use [91]. These findings demonstrate that concerns over antibiotic residues and resistant bacteria have a strong influence on purchase decisions. Hence, Hypotheses 5 and 6 are as follows:

**H5:**  *Concerns over antibiotic residues will positively affect purchase intention towards meat produced without preventive antibiotic use*.

**H6:**  *Concerns over antibiotic-resistant bacteria will positively affect purchase intention towards meat produced without preventive antibiotic use*.

#### 1.1.5. Attitude Towards Preventive Antibiotic Use in Food Animals

According to the TPB, attitude is a main driver of the intention to perform a behavior [60]. Indeed, numerous studies have demonstrated the link between attitude and purchase intentions [66,92,93,94,95]. For instance, a favorable attitude towards organic food has a strong positive effect on consumers’ purchase intention and willingness to pay more for organic food [66,74,80]. Emerging research suggests that a negative attitude towards antibiotic use in food animals leads to a higher intention to buy or willingness to pay for food produced with reduced antibiotic use or antibiotic free [42,61,96]. Accordingly, it is very likely that consumers’ attitude towards preventive antibiotic use is a key factor affecting their purchase intention towards meat produced without it. Therefore, Hypothesis 7 is as follows:

**H7:**  *A negative attitude towards preventive antibiotic use in food animals will positively affect purchase intention towards meat produced without preventive antibiotic use*.

In summary, the present study aims to examine consumers’ purchase intention towards meat produced without preventive antibiotic use and the influencing factors. Informed by the TPB and extended TPB frameworks as well as the relevant literature, we hypothesize that this purchase intention will be driven by various factors, including health consciousness, trust in responsible antibiotic use in farming, objective knowledge about antibiotics used in food animals, subjective knowledge about preventive antibiotic use, concerns over antibiotic residues and resistant bacteria, and attitudes towards preventive antibiotic use.

## 2. Materials and Methods

### 2.1. Survey Design and Procedure

To investigate the nature and predictors of consumers’ purchase intention towards meat produced without preventive antibiotic use, a cross-sectional survey was conducted in China. The survey was developed in English and translated into Chinese by two bilingual researchers (Y.Z. and A.Z.). The study was conducted in compliance with the ethical standards specified in the Australian National Statement on Ethical Conduct in Human Research 2007 (updated 2018) [97]. Ethical approval was obtained from the Social Science Human Research Ethics Committee of Commonwealth Scientific and Industrial Research Organisation (CSIRO) (approval number: 093/23). The online survey was administered via a professional research platform (Credamo). Participants who were 18 years old or older were eligible for participation. The survey link was randomly distributed to eligible subjects within the platform’s database of 3 million participants. Interested participants read the informed consent form first. In particular, they were informed that the participation is voluntary and that they can withdraw at any time. Participants were also informed that no personally identifiable information would be collected and that all data would be handled confidentially. To indicate their consent to participate in the survey, participants were instructed to click the ‘Next page’ button. After completing the survey, participants received a small amount of money for their time.

The survey questionnaire consisted of three sections. The first section contained demographic questions (i.e., gender, age group, and education). The remaining two sections consisted of questions specifically aimed at testing the seven hypotheses outlined in the Introduction section. The second section included questions measuring independent variables outlined in Hypotheses 1–4, including health consciousness, trust in responsible antibiotic use in farming, objective knowledge about antibiotics used in food animals, and subjective knowledge about preventive antibiotic use. The last section consisted of questions assessing the independent variables outlined in Hypotheses 5–7 (i.e., concerns over antibiotic residues and resistant bacteria, and attitude towards preventive antibiotic use), and the dependent variable (purchase intention towards meat produced without preventive antibiotic use).

Considering that consumers might have little knowledge about antibiotic resistance and preventive antibiotic use in food animals [41], the following definitions were provided to all participants before the last section of the survey:

Antibiotic and antibiotic resistance

- *Antibiotics are used to treat bacterial infections in animals and humans.*
- *Antibiotic resistance means bacteria, not animals or humans, become resistant to antibiotics. This makes antibiotics become less effective in treating certain bacterial infections.*

The preventive antibiotic use in food animals

- *Antibiotics are often fed to healthy animals to prevent them from getting sick. For this purpose, antibiotics are often added to animal feed or water.*

The provided information was adapted from previous research [91]. Providing this background information enabled participants to understand and answer the questions in the last section of the survey. To make sure participants read it attentively, the information was presented on the page for 45 s before participants could move on to the next section.

### 2.2. Measures

A 5-point Likert scale (1 = strongly disagree, 5 = strongly agree; unless stated otherwise) was applied to all measured variables except for knowledge. For variables measured with multiple items, the average scores of items were used in the data analysis. The reliability of the measures was assessed using Cronbach’s alpha. An α value of 0.60 or higher was considered acceptable, particularly when the number of items was low [98].

*Health consciousness* was measured with 3 items adapted from Pham et al. [99] and Chen [100]: “I choose food carefully to ensure good health”, “I think of myself as a health-conscious person”, and “I always take good care of my health” (α = 0.65).

*Trust in responsible antibiotic use* was measured with 3 items adapted from Zhang et al. [101]: “I trust that farmers use antibiotics responsibly with their food animals”, “I trust that farmers only use antibiotics with their food animals when they are sick”, and “I trust that farmers strictly follow the regulations on antibiotic use in their farming practice” (α = 0.83).

*Objective knowledge* was assessed by 4 items adapted from Meerza et al. [22]. Participants were asked to indicate if they think each statement is true, false, or if they do not know. The items were: “Antibiotics can be used to treat bacterial infections in food animals” (True), “Antibiotics can be used to treat viral infections in food animals” (False), “Antibiotics can be used to treat any kind of inflammation in food animals” (False), and “Antibiotics used for treating food animals are different from those used for treating humans” (False). The number of correct answers was used to reflect participants’ objective knowledge.

*Subjective knowledge.* Participants were asked if they have heard of preventive antibiotic use in food animals (Yes/No). Participants who answered yes were further asked to indicate how much they know about it on a 5-point scale (1 = I don’t know at all, 5 = I know a lot). To facilitate data analysis, for participants who reported they had not heard of preventive antibiotic use, the score of subject knowledge was coded into 1 (I don’t know at all).

*Concerns over antibiotic residues* were assessed by 4 items adapted from Michaelidou and Hassan [70] (e.g., “I’m concerned about the amount of antibiotic residues in meat”, “I’m concerned about the amount of antibiotic residues in the environment”, α = 0.74).

*Concerns over antibiotic-resistant bacteria* were assessed by 4 items adapted from Michaelidou and Hassan [70] (e.g., “I’m concerned about the amount of antibiotic-resistant bacteria in meat”, “I’m concerned about the amount of antibiotic-resistant bacteria in the environment”, α = 0.78).

*Attitude towards preventive antibiotic use* was assessed by two items adapted from Meerza et al. [22] and Lusk et al. [102]. Participants were asked to indicate to what extent they accept preventive antibiotic use in food animals (1 = totally unacceptable, 5 = perfectly acceptable; reverse coded), and to what extent they support a ban on preventive antibiotic use in food animals (1 = I don’t support at all, 5 = I totally support) (α = 0.67). A higher average score indicated a more negative attitude towards preventive antibiotic use.

*Purchase intention* towards meat produced without preventive antibiotic use was assessed by two items adapted from Bradford et al. [61]: “I intend to buy meat produced without preventive antibiotic use” and “I will look for meat produced without preventive antibiotic use” (α = 0.75).

*Demographics*: Existing research suggested that certain demographic variables, such as gender, age, and education, were associated with consumers’ purchase intention towards food produced with reduced antibiotic use or antibiotic-free food [40,103,104]. Therefore, we included these socio-demographic characteristics as control variables in this study. To facilitate statistical analysis, demographic variables were coded numerically. Gender was coded as 1 = male, 2 = female. Age groups were categorized using commonly applied age brackets, e.g., [42,61,105,106,107]: 1 = 18–24 years (the minimum eligible age for participation was 18); 2 = 25–34 years; 3 = 35–44 years; 4 = 45–54 years; 5 = 55+ years. Education levels were coded as 1 = senior high school and below (year 12); 2 = college certificate; 3 = bachelor’s degree; 4 = postgraduate.

### 2.3. Data Analysis

SPSS version 22.0 was used for data analysis. Descriptive statistical analysis and Pearson correlation analysis were conducted to explore measured variables and their correlations. Hierarchical multiple regression analysis was implemented to test hypotheses and identify key factors influencing purchase intention. Results were considered statistically significant if *p* < 0.05.

## 3. Results

### 3.1. Participants’ Demographic Characteristics

A total of 1336 participants completed the survey. After excluding invalid samples, identified by quality-control trap questions embedded in the survey, 1123 valid samples (84.1%) were used for data analysis.

Table 1 presents the participants’ demographic characteristics. Most participants were female (62.1%), between 18 and 44 years old (88.7%), and with an education level of a bachelor’s degree or higher (82.1%).

### 3.2. Descriptive Statistics and Correlations Between Measured Variables

#### 3.2.1. Descriptive Statistics of Measured Variables

**Figure 1:**
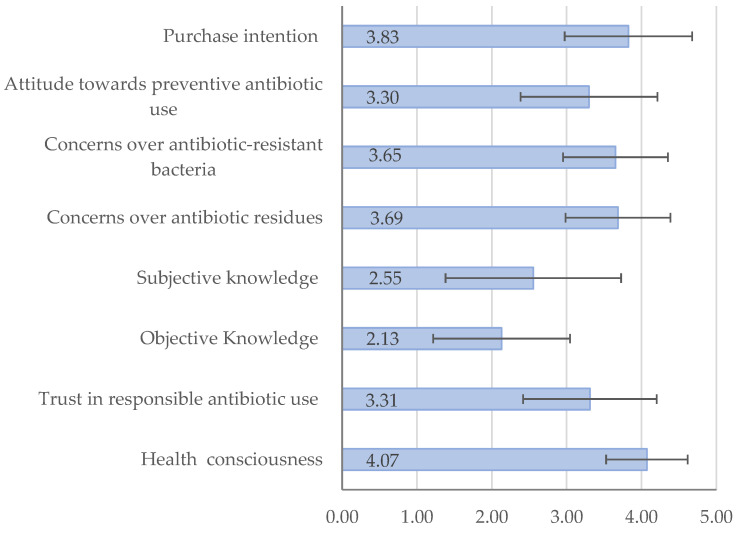
Mean scores of measured variables with standard deviations. Note: Objective knowledge was assessed by the number of correct answers of four true–false questions, with a score range of 0–4. All other variables were measured on a 5-point Likert scale (e.g., 1 = strongly disagree, 5 = strongly agree).

Figure 1 presents the mean scores and standard deviations of the measured variables. The mean score of health consciousness was above 4 out of a possible 5, suggesting that participants were self-conscious about their health. The mean score of trust in responsible antibiotic use was slightly above the neutral value of 3, indicating that participants had some trust in responsible antibiotic use, but the trust level was not very high.

On average, participants correctly answered 2.13 out of 4 questions regarding objective knowledge about antibiotics used in food animals. Although most participants correctly recognized that antibiotics can treat bacterial infections in food animals (83.3%) but cannot treat all types of inflammation (76.9%), only 44.2% of them realized that antibiotics cannot treat viral infections. Furthermore, 79.8% participants wrongfully believed that antibiotics used in food animals are different from those used in humans. The mean score of subjective knowledge was lower than the neutral value of 3, with 29.7% participants reporting they had not heard of preventive antibiotic use in food animals before.

On average, participants had some concerns over antibiotic residues and resistant bacteria. The mean score of attitude was slightly above the neutral value of 3, indicating that participants had an overall negative attitude towards preventive antibiotic use, but it was not very strong. Noticeably, 56.5% participants expressed some degree of support for a ban on preventive antibiotic use. Finally, participants expressed a strong intention to buy meat produced without preventive antibiotic use.

#### 3.2.2. Correlations Between Measured Variables and Demographic Variables

Table 2 presents Pearson correlations between the measured variables and the demographic variables. Age was positively correlated with all measured variables, except for objective knowledge, indicating that older participants had a higher score in these variables. Gender was not significantly related to any of the measured variables. Education was only positively correlated with objective knowledge, suggesting that participants with higher education had better knowledge about antibiotics used in food animals.

Health consciousness was positively related to all measured variables, except for objective knowledge. Trust in responsible antibiotic use was positively correlated with subjective knowledge. However, trust was negatively related to concerns over antibiotic residues and resistant bacteria, as well as attitude towards preventive antibiotic use, suggesting that a higher level of trust was linked to less concerns and a more positive attitude towards preventive antibiotic use. Objective knowledge and subjective knowledge were positively but weakly correlated (*r* = 0.15).

Concerns over antibiotic residues, concerns over antibiotic-resistant bacteria, and attitude towards preventive antibiotic use were positively correlated with each other. In particular, a strong positive relationship (*r* = 0.79) between concerns over antibiotic residues and concerns over antibiotic-resistant bacteria was observed, indicating that these two variables were highly correlated. However, multicollinearity diagnostics found that multicollinearity was not a serious concern in this study. Multicollinearity diagnostics was conducted by examining the variance inflation factor (VIF) and tolerance statistics. A VIF value greater than 5 and a tolerance value less than 0.10 were considered indicative of significant multicollinearity [108,109]. The analysis revealed that the VIF values for concerns over antibiotic residues and resistant bacteria were 2.78 and 2.67, respectively, and the tolerance values were 0.36 and 0.38, respectively.

Finally, all measured variables were positively linked to purchase intention towards meat produced without preventive antibiotic use.

### 3.3. Predicting Purchase Intention Towards Meat Produced Without Preventive Antibiotic Use

A hierarchical multiple regression analysis was conducted to identify key variables affecting purchase intention and to test the proposed hypotheses. Demographics were entered as control variables in step 1 (model 1). All measured variables were entered in step 2 (model 2).

Table 3 presents the results of the regression analysis. In model 1, demographic variables explained 4% of the variance in purchase intention (based on *R_adj_*^2^), with age and education being significant predictors. That is, participants who were older and had higher education reported a greater purchase intention. After controlling for demographics and introducing proposed affecting factors in model 2, the explanatory variance of the predictors increased by 23%. Moreover, no demographics were significantly related to purchase intention anymore.

Health consciousness was positively associated with purchase intention, indicating that more health-conscious participants expressed a greater intention to buy meat produced without preventive antibiotic use. Thus, H1 was supported. Trust in responsible antibiotic use was positively linked to purchase intention, suggesting that a higher level of trust led to a greater purchase intention. Hence, H2 was supported. Both objective and subjective knowledge were significant predictors of purchase intention. Such that H3 and H4 were supported. Concerns over antibiotic residues were positively associated with purchase intention, providing support for H5. Concerns over antibiotic-resistant bacteria, however, were not significantly related to purchase intention. That is, H6 was not supported by the results. Lastly, in support of H7, attitude towards preventive antibiotic use was a significant driver of purchase intention.

Among these significant predictors, attitude was the most influential (*β* = 0.35), followed by health consciousness (*β* = 0.12), concerns over antibiotic residues (*β* = 0.12), and trust in responsible antibiotic use (*β* = 0.11); while objective knowledge (*β* = 0.06) and subjective knowledge (*β* = 0.08) were the least influential.

## 4. Discussion

Antibiotics are widely used in healthy animals to prevent diseases, which is contributing to the rising threat of antibiotic resistance [29,30,31,32]. This study sets out to explore consumers’ purchase intention towards meat produced without preventive antibiotic use and the key factors driving this purchase intention. This research found that consumers had a strong purchase intention towards meat produced without preventive antibiotic use, indicating a great potential market for these food products. This finding is particularly valuable for stakeholders who strive to promote responsible antibiotic use in the current context, where increasing concerns over overall antibiotic use in food animals have facilitated the demand for food produced without any use of antibiotics, which may deny farmers the opportunity to treat sick animals and maintain their welfare [42,43,44,45,46]. Further, previous studies suggested that although having a great intention to buy food produced with reduced antibiotic use or antibiotic-free food, consumers were only willing to pay a small premium for these products [40,61,90,110]. These findings suggest that when promoting meat produced without preventive antibiotic use, livestock industries should strike a balance between achieving profitability for farmers and keeping price affordable for consumers.

Attitude was found to be the most influential predictor of consumers’ purchase intention in the current study. Consumers who held a stronger negative attitude towards preventive antibiotic use expressed a higher intention to buy meat produced without it. A correlation analysis indicated that attitude was closely related to concerns over antibiotic residues and resistant bacteria. That is, consumers with higher risk perceptions of antibiotic residues and resistant bacteria held a stronger negative attitude towards preventive antibiotic use. Previous research found consumers’ support for a ban on preventive antibiotic use largely increased after exposure to risk information about it [91]. These results suggest that enhancing consumers’ understanding of the health risks of preventive antibiotic use is an effective strategy to shape their attitude.

Health consciousness was an important predictor of consumers’ purchase intention towards meat produced without preventive antibiotic use. This is consistent with previous literature showing that health-conscious consumers were more likely to consume organic food as they considered these products to be healthier and free of harmful chemicals and additives [70,71,72,74,75,76].

In line with previous research [66,75,77,78,79], the current study found that trust in responsible antibiotic use was an important predicting factor of purchase intention. However, consumers in this study only had limited trust that antibiotics are used responsibly and in compliance with regulations in farming. Low levels of trust were also found in previous studies, where consumers were skeptical about the justification of antibiotic use in food animals [43] and believed that not enough regulations were in place to ensure responsible antibiotic use in farming [41,103,111]. These findings indicate that it is necessary to build consumers’ trust first when promoting meat produced without preventive antibiotic use. Moreover, the correlation analysis revealed that a higher level of trust in responsible antibiotic use was linked to a lower level of concerns over antibiotic residues and resistant bacteria. This result suggests that fostering consumers’ trust in responsible antibiotic use may alleviate excessive concerns about all types of antibiotic usage in food animals, thus helping to address the increasing demand for food produced without any use of antibiotics.

Both objective and subjective knowledge were significant predictors of consumers’ purchase intention. These results are consistent with the existing literature investigating the impact of knowledge on purchase decisions of various products (e.g., environmentally sustainable food, imported functional food, and green products) [85,86,87]. The present study found that both objective and subjective knowledge were limited, highlighting the need to enhance consumers’ knowledge through increased public communication and engagement. Moreover, objective and subjective knowledge had a weak relationship, which is in line with a previous study on the correlation between objective and subjective knowledge of organic cotton apparel [81]. These findings demonstrate that objective and subjective knowledge are different measures of knowledge. Therefore, future studies need to take both types of knowledge into consideration when examining the effect of knowledge on purchase decisions.

Interestingly, consumers’ purchase intention was only significantly affected by concerns over antibiotic residues, but not by concerns over antibiotic-resistant bacteria. This might be due to the abstract nature of antibiotic resistance, which has led to misunderstanding and misconceptions among consumers [41,91]. In contrast, antibiotic residues are more directly linked to food, and their health risks are more straightforward for consumers. The results indicate that when educating the public about antibiotic resistance, the concept may need to be more clearly explained. For example, using terms such as “super bugs”, it is much easier for consumers to understand the concept and transmission risk of antibiotic-resistant bacteria [41].

Overall, the results provided support for most of our hypotheses, except for H6. While this may make the findings seem predictable, it is important to note that the development of the hypotheses was heavily based on a theoretical framework that is widely adopted in consumer research (the Theory of Planned Behavior) and the literature that is highly relevant to our study. The predictability of the results may also highlight the shared underlying factors shaping consumers’ food purchase decisions, particularly around food safety concerns. Such concerns could stem from increased health literacy, a general mistrust of the food production system, and an increasing trend of aversion to chemicals and food additives. Recognizing these shared causes provides insights into the core factors driving consumer food choices, enabling more targeted interventions to address food safety concerns and influence purchase behaviors.

To the best of our knowledge, this is the first attempt to investigate the drivers of consumers’ behavioral intention towards preventive antibiotic use in food animals. The study contributes to the literature in two ways. First, with regard to consumers’ perspectives on antibiotic use in food animals, most previous studies have focused on overall antibiotic use without distinguishing the purpose of usage, e.g., [43,82,90,103,111,112,113,114], which might have caused confusion among research participants and led to conflicting findings. To address this issue, this study specifically focused on the use of antibiotics for disease prevention. Second, the current study advances the literature by examining what motivates consumers to choose meat free from preventive antibiotics, adding depth to the emerging body of research on antibiotic-free food products. This is especially valuable, as marketing for antibiotic-free food products may mislead consumers to believe that all antibiotic usage is harmful.

The findings of this study can provide deep insights for policymakers and livestock industries who seek to develop effective strategies to restrict preventive antibiotic use and to increase consumers’ purchase intention towards meat produced without it. First, this study reveals a negative attitude towards preventive antibiotic use in food animals and a strong purchase intention towards meat produced without it among consumers. In particular, most consumers expressed some degree of support for a ban on it, which may challenge livestock industries’ social license to operate. These empirical findings on consumers’ demands can provide grounds for policymakers and livestock industries to take measures to address this practice. For instance, in response to this consumer demand, and following the EU’s lead [17] as well as the WHO’s recommendation [18], governments can introduce legislation to restrict and govern antibiotic use for disease prevention in food animals. In addition, policymakers and livestock industries should focus on effective communication with consumers through information disclosure and transparency (i.e., implementing a traceability system). This will help inform consumers about how antibiotics are used, and what measures have been taken to ensure responsible use and to prevent antibiotic residues and resistant bacteria in food (e.g., withdrawal periods). Such a measure will help build consumers’ trust. Second, the present study suggests that consumers had limited knowledge about preventive antibiotic use. Policymakers and livestock industries can enhance public communication and engagement to increase consumers’ understanding about how antibiotics are used in food animals and the health hazards of preventive use. Further, given that increasing consumers’ health consciousness might be an effective way to enhance purchase intention, future communications could aim to improve consumers’ overall health consciousness.

While this research provides valuable insights into the factors driving consumers’ purchase intention towards meat produced without preventive antibiotic use, it has several limitations. First, the cross-sectional design limited the ability to infer causality between the identified predictors and purchase intention. Future research should employ longitudinal designs to assess how these factors affect consumers’ purchase intention over time. Second, although we adopted the Theory of Planned Behavior as the theoretical basis, we did not include perceived social norm or perceived behavioral control in this study due to the hypothetical nature of meat produced without preventive antibiotic use. Future studies can explore ways to incorporate the full framework or consider alternative theories (e.g., the Health Belief Model) to further understand the psychological factors driving purchase intention. Third, the low correlation between objective and subjective knowledge observed in this study could be due to the measures used, where they measured different aspects of knowledge. Objective knowledge mainly focused on antibiotics used in food animals, whereas subjective knowledge was primarily about preventive antibiotic use. To further identify the relationship between two types of knowledge and their impacts on purchase decisions, future research needs to measure objective and subjective knowledge on the same aspect of antibiotic use in food animals. Lastly, most participants in this study were from a highly educated, younger population. Future research can investigate the identified predictors in various demographic groups and geographic settings to enhance the generalizability of the results.

## 5. Conclusions

This study highlights a strong consumer purchase intention towards meat produced without preventive antibiotic use, suggesting that restricting preventive antibiotic use aligns with consumer demand. Further, the study outlines the key factors driving the purchase intention, including health consciousness, trust in responsible antibiotic use in farming, objective and subjective knowledge about the use of antibiotics in food animals, concerns over antibiotic residues, and attitude towards preventive use of antibiotics. For policymakers and livestock industries who seek to restrict preventive antibiotic use and promote meat produced without it, public communications should focus on enhancing consumers’ understanding of preventive antibiotic use and the health risks associated with it (i.e., increasing the transmission risk of antibiotic-resistant bacteria). Simultaneously, stricter regulatory measures may be introduced to govern preventive antibiotic use and increase transparency in the process.

## Figures and Tables

**Table 1:** Participants’ demographic characteristics.

Variables	*N*	Percentage (%)
Gender		
Male	426	37.9
Female	697	62.1
Age		
18–24 years	370	32.9
25–34 years	459	40.9
35–44 years	167	14.9
45–54 years	76	6.8
55+ years	51	4.5
Education		
Senior high school and below (year 12)	83	7.4
College certificate	118	10.5
Bachelor’s degree	739	65.8
Postgraduate	183	16.3

**Table 2:** Pearson correlations between measured variables and with demographics.

Variable	Age	Gender	Education	Health Consciousness	Trust in Responsible Antibiotic Use	Objective Knowledge	Subjective Knowledge	Concerns over Antibiotic Residues	Concerns over Antibiotic-Resistant Bacteria	Attitude Towards Preventive Antibiotic Use
Health consciousness	0.24 ***	−0.02	0.01							
Trust in responsible antibiotic use	0.15 ***	−0.00	0.00	0.30 ***						
Objective knowledge	0.00	0.03	0.12 ***	0.02	−0.06					
Subjective knowledge	0.17 ***	−0.03	0.04	0.24 ***	0.20 ***	0.15 ***				
Concerns over antibiotic residues	0.10 **	0.05	−0.02	0.15 ***	−0.16 ***	0.03	0.03			
Concerns over antibiotic-resistant bacteria	0.07 *	0.05	−0.05	0.13 ***	−0.12 ***	0.04	0.01	0.79 ***		
Attitude towards preventive antibiotic use	0.22 ***	0.05	−0.04	0.15 ***	−0.11 ***	0.04	−0.01	0.40 ***	0.37 ***	
Purchase intention	0.19 ***	−0.02	0.02	0.27 ***	0.10 **	0.09 **	0.15 ***	0.31 ***	0.28 ***	0.44 ***

Note: * *p* < 0.05, ** *p* < 0.01, *** *p* < 0.001. Gender: 1 = male, 2 = female. Age: 1 = 18–24 years; 2 = 25–34 years; 3 = 35–44 years; 4 = 45–54 years; 5 = 55+ years. Education: 1 = senior high school and below (year 12); 2 = college certificate; 3 = bachelor’s degree; 4 = postgraduate. Objective knowledge was assessed by the number of correct answers of four true–false questions, with a score range of 0–4. All other variables were measured on a 5-point Likert scale (e.g., 1 = strongly disagree, 5 = strongly agree).

**Table 3:** Standardized regression coefficients (*β*) from regression analysis predicting participants’ purchase intention towards meat produced without preventive antibiotic use, testing the effects of demographic variables only (model 1) and all variables (model 2).

	Model 1		Model 2	
	*β*	*t*	*β*	*t*
Age	0.21	7.03 ***	0.05	1.88
Gender	−0.01	−0.48	−0.04	−1.68
Education	0.07	2.46 *	0.04	1.63
Health consciousness			0.12	4.30 ***
Trust in responsible antibiotic use			0.11	3.78 ***
Objective knowledge			0.06	2.24 *
Subjective knowledge			0.08	2.81 **
Concerns over antibiotic residues			0.12	2.81 **
Concerns over antibiotic-resistant bacteria			0.05	1.25
Attitude towards preventive antibiotic use			0.35	−12.28 ***
Model Summary				
* R* ^2^	0.04		0.28	
* R_adj_* ^2^	0.04		0.27	
* R*^2^ change			0.23	

Note: * *p* < 0.05, ** *p* < 0.01, *** *p* < 0.001. Gender: 1 = male, 2 = female. Age: 1 = 18–24 years; 2 = 25–34 years; 3 = 35–44 years; 4 = 45–54 years; 5 = 55+ years. Education: 1 = senior high school and below (year 12); 2 = college certificate; 3 = bachelor’s degree; 4 = postgraduate. Objective knowledge was assessed by the number of correct answers of four true–false questions, with a score range of 0–4. All other variables were measured on a 5-point Likert scale (e.g., 1 = strongly disagree, 5 = strongly agree).

## Data Availability

The data used in this study are available from the corresponding author upon request.
